# The Differential Antitumor Activity of 5-Aza-2'-deoxycytidine in Prostate Cancer DU145, 22RV1, and LNCaP Cells

**DOI:** 10.7150/jca.56709

**Published:** 2021-07-25

**Authors:** Huiying Cheng, Sijie Tang, Xueqi Lian, Hong Meng, Xiang Gu, Jiajia Jiang, Xiaohua Li

**Affiliations:** 1 Aoyang Institute of Cancer, Affiliated Aoyang Hospital of Jiangsu University, 279 Jingang Blvd., Zhangjiagang, Suzhou, 215600, China; 2 Dept of Urology, the Affiliated Aoyang Hospital of Jiangsu University, 279 Jingang Blvd., Zhangjiagang, Suzhou, 215600, China; 3 Perinatology Research Branch, Eunice Kennedy Shriver National Institute of Child Health and Human Development, National Institutes of Health, Detroit 48201, MI, USA; 4 The Laboratory of Clinical Genomics, Hefei KingMed Diagnostics Ltd., 2800 Chuangxin Blvd., Building H4, Hefei 230088, China; 5 National Center for Gene Testing Technology Application & Demonstration(Hefei), 2800 Chuangxin Blvd., Building H4, Hefei 230088, China

**Keywords:** 5-Aza-2′-deoxycytidine, prostate cancer cell, antitumor activity, p53, p21

## Abstract

DNA methylation is a DNA methyltransferase-mediated epigenetic modification affecting gene expression. This process is involved in the initiation and development of malignant disease. 5‐Aza‐2′‐deoxycytidine (5‐Aza), a classic DNA methyltransferase inhibitor, possesses antitumor proliferation activity. However, whether 5-Aza induces cytotoxicity in solid tumors warrants further investigated. In this study, human prostate cancer (CaP) cells were treated with 5-Aza and subjected to cell viability and cytotoxicity analysis. Reverse transcription-polymerase chain reaction and methylation-specific polymerase chain reaction assay were utilized to test the gene expression and methylation status of the p53 and p21 gene promoters. The results showed that 5-Aza differentially inhibited spontaneous proliferation, arrested the cell cycle at S phase in DU145, at G1 phase in 22RV1 and LNCaP cells, and G2 phase in normal RWPE-1 cells, as well as induced the expression of phospho-H2A.X and tumor suppressive mammary serine protease inhibitor (maspin) in all three types of CaP cells. 5-Aza also increased p53 and p21 transcription through promoter demethylation, and decreased the expression of oncogene c-Myc in 22RV1 and LNCaP cells. Western blotting analysis showed that the poly (ADP-ribose) polymerase cleavage was detected in DU145 and 22RV1 cells. Moreover, there were no significant changes in p53, p21 and c-Myc expression in DU145 cells following treatment with 5-Aza. Thus, in responsible for its apoptotic induction and DNA damage, the mechanism of the antitumor activities of 5-Aza may involve in an increase of tumor suppressive maspin, upregulation of wild type p53-mediated p21 expression and a decrease of oncogene c-Myc level in 22RV1 and LNCaP cells, and enhancing the tumor suppressive maspin expression in DU145 cells. These results enriched our understanding of the multifaceted antitumor activity of 5-Aza, and provided the expression basis of biomarkers for its possible clinical application in prostate cancer.

## Introduction

DNA methylation is an important epigenetic modification that regulates gene expression and plays an important role in mammalian development. This biological chemical modification is mediated by DNA methyltransferases (DNMTs), including DNMT1, DNMT3A and DNMT3B, eventually adding a methyl group to a CpG dinucleotide island of DNA nucleotides by covalent bonding 1. In human cancers, studies found that the abnormal hypermethylation of a gene promoter region was associated with the silencing of gene expression, typically tumor suppressor genes, and led to cellular malignant transformation and tumorigenesis. Meanwhile, accumulating data suggested that targeting DNA methylation could be a useful strategy for the treatment of cancer 2, 3. Currently, a few hypomethylating agents (HMAs) are available for research and clinical investigation, e.g., 5-Aza-2′-deoxycytidine (5-Aza, decitabine) and 5-Azacytidine (5-Aza-CR). These agents functioned as DNMT inhibitors and markedly restored the silenced gene expression 4.

5-Aza and 5-Aza-CR were approved by the United States Food and Drug Administration in 2006 and 2004, respectively, for the treatment of myelodysplastic syndromes and other hematologic malignancy diseases 5. In fact, 5-Aza has stronger demethylation ability compared with 5-Aza-CR 6. Studies in *in vitro* and animal models of human cancer suggested that 5-Aza exerted a greater therapeutic effect than 5-Aza-CR. Two major mechanisms have been proposed for its anti-tumor cytotoxicity 7-9. On one hand, 5-Aza reactivated the silenced expression of tumor suppressive genes by its demethylation activity on cellular DNA; on the other hand, it induced double-strand DNA damage due to the formation of irreversible, covalent 5-Aza/DNMTs/DNA adducts, thereby depleting the cells with abnormal DNMT activity. However, 5-Aza is less effective in the treatment of solid tumors 10. It was proposed that there may be different tumor microenvironments and/or multiple key factors affecting and regulating the therapeutic activity of 5-Aza in different cell types.

The p53, a classic tumor suppressor gene, plays an important role in cell biology including cell cycle process, DNA repair, senescence, and induction of ferroptosis and apoptosis 11, 12. Meanwhile, p21^WAF1/Cip1^ is a critical downstream factor of the p53 gene and functions as a cyclin-dependent kinase inhibitor to arrest the cell cycle at the G1 or G2 phase in favor of DNA damage repair etc. 13. In addition, oncogene c-Myc has also been found to be associated with cell cycle process and be negatively regulated by p53 14-16. Both mRNA and protein levels of c-Myc are increased in a variety of human malignant diseases, e.g., breast, gastric, colorectal cancers, etc. 17, 18. In addition, overexpression of c-Myc is associated with the promotion of proliferation, angiogenesis, metastasis, and early tumor recurrence 19.

Prostate cancer (CaP), a common type of malignant tumors, is an important cause of cancer-related death in men worldwide 20. Abnormal DNA methylation (hypermethylation and hypomethylation) is the most characteristic epigenetic change of this disease, and has been associated with the initiation and development of prostate cancer 21-23. Numerous studies reported that 5-Aza exerted antitumor activity by activating p53/p21, and a few related mechanisms have been investigated in prostate cancer 24-27. However, some conflicting results were also reported. For example, utilizing prostate tumor cells and mouse models, studies have shown that 5-Aza induced tumorigenesis by promoting epithelial mesenchymal transition and epithelial dedifferentiation for the formation of tumor stem cells 28. Thus, clarifying the antitumor activity of 5-Aza in prostate cancer is desirable for research purposes and its clinical application. In the present study, to explore how the expression of the tumor suppressive maspin, p53, p21 and oncogene c-Myc were involved in 5-Aza-induced anti-tumor activity, the effects of 5-Aza on cell proliferation, cell cycle process, DNA damage, and apoptosis were investigated in different grade of human CaP epithelial cell lines, including DU145 (androgen independent metastatic carcinoma cell), 22RV1 ( a CaP cell derived from CWR22 xenograft with weak androgen-dependence feature), LNCaP (androgen-sensitive metastatic carcinoma cell), and RWPE-1 (normal immortalized nonmalignant human prostate epithelial cell). Furthermore, the 5-Aza- induced demethylation of the p53 and p21 gene promoters, along with c-Myc expression, were analyzed and discussed.

## Materials and Methods

### Cell Culture and Treatments

Normal immortalized human prostate epithelial RWPE-1 cells were obtained from Beijing Beina Chuanglian Biotechnology Institute (Beijing, China) and cultured in Keratinocyte Medium with keratinocyte growth supplement and 1% penicillin/streptomycin (ScienCell Research Laboratories, USA). CaP cell lines DU145, 22RV1, and LNCaP were purchased from the cell bank of the Chinese Academy of Sciences (Shanghai, China) and cultured in a humidified incubator with 6% CO2 at 37°C. DU145 cells were maintained in Dulbecco's modified Eagle's medium (DMEM; Gibco, Shanghai, China) supplemented with 10% fetal bovine serum (FBS; Biological Industries, Israel) and 1% penicillin/streptomycin (Gibco) according to the instructions provided by the manufacturer. 22RV1 and LNCaP cells were maintained in RPMI 1640 medium (Gibco) with the same additives as above. All the cells were negative for mycoplasma-tested using the LookOut^®^ Mycoplasma PCR Detection Kit (Sigma-Aldrich, St. Louis, MO, USA). For the drug treatment study, the cells were treated with fresh medium containing 5-Aza (purity ≥ 97%, Sigma-Aldrich) and the medium was replaced every 24 h for up to 96 h.

### WST-1 Assay

Cell viability was measured using the WST-1 assay. Briefly, cells were seeded in 96-well plates at 4,000 cells per well, and treated with different concentrations of 5-Aza in triplicate for an indicated period of time. At the end of the treatment, 20 μl of WST-1 solution (Beyotime Biotechnology, Shanghai, China) was added into each well and the cells were cultured at 37°C for an additional 2 h. Finally, the absorbance was measured at 450 nm and 690 nm using a microplate reader (Thermo Fisher Scientific, Vantaa, Finland). The data were calculated by optical density (OD) OD450-OD690 and represented the cell viability.

### 2.3 Trypan Blue Staining Assay

2 × 10^5^ RWPE-1 cells, 2.5 × 10^5^ DU145 cells and 3 × 10^5^ 22RV1/LNCaP cells were seeded in six-well plates. Following treatment with 5-Aza (2 μM, 4 μM and 8 μM) and incubation for 96 h, the cells were trypsinized. Then the cell suspension was mixed 1:1 with Trypan blue solution (Beyotime Biotechnology), and observed cell morphology under a light microscope (Nikon Corporation, Tokyo, Japan) within 5 min.

### Flow Cytometry Assay

For the cell cycle status analysis, 2 × 10^5^ RWPE-1 cells, 2.5 × 10^5^ DU145 cells and 3 × 10^5^ 22RV1/LNCaP cells were seeded in six-well plates. Following treatment with 5-Aza (2 μM) and incubation for 96 h, the cells were harvested and fixed in 70% ethanol overnight. Subsequently, the cells were washed with ice cold phosphate-buffered saline and stained with propidium iodide (PI) solution containing RnaseA (Beyotime Biotechnology) for 30 min at 37°C. The stained cells were analyzed using a flow cytometer (Beckman Coulter Life Sciences, Brea, CA, USA), and the cell DNA content for cell cycle distribution was analyzed with the flowjo 7.6 software. All measurements of each cell were performed with the same instrument settings.

For the apoptotic cell death analysis, cells (2.5 × 10^5^) were seeded into six-well plates and treated with 5-Aza at 2 µM for 96 h. Next, the cells were digested with trypsin without ethylene diamine tetraaceticacid, washed twice in ice cold phosphate-buffered saline, and stained with the fluorescein isothiocyanate (FITC) labeled Annexin V and PI kit (BD Biosciences, Franklin Lakes, NJ, USA) according to the instructions provided by the manufacturer. The stained cells were analyzed using a flow cytometer (Beckman Coulter Life Sciences). The apoptotic cells with Annexin V-FITC staining were calculated. All measurements of each cell were performed with the same instrument settings.

### Western Blotting Assay

Cells were lysed in radio immunoprecipitation assay buffer (Nobleryder, Beijing, China) with 1 mM of phenylmethanesulfonyl fluoride. Next, the protein concentration was measured using the BCA Protein Assay Kit (Thermo Scientific, Rockford, AL, USA). Equal total amounts of protein samples were loaded and the proteins were separated using 10% or 12% sodium dodecylsulfate-polyacrylamide gel electrophoresis gels. Subsequently, the proteins were transferred onto polyvinylidene difluoride membranes (Millipore, Bedford, MA, USA). For the immunoblotting assay, the membrane was blocked with 5% nonfat milk for 40 min and incubated with primary antibodies for 2 h at room temperature. The primary antibodies of anti-poly (ADP-ribose) polymerase (anti-PARP) and anti-phospho-H2A.X (Ser139) were obtained from Cell Signaling Technology (Danvers, MA, USA). Anti-DNMT1, anti- mammary serine protease inhibitor (anti-maspin), anti-c-Myc, anti-p53, and anti-p21 were purchased from Abcam. The membranes were then incubated with horseradish peroxidase-conjugated anti-mouse IgG secondary antibody (Abcam, Cambridge, UK) or horseradish peroxidase-conjugated anti-rabbit IgG secondary antibody (Abcam) for 1 h at room temperature. Finally, protein expression was detected by the enhanced chemiluminescence system and examined using Tanon 5500 (Tanon, Shanghai, China).Data was obtained by normalizing the protein levels to those of glyceraldehyde-3-phosphate dehydrogenase (GAPDH) (Abcam).

### RNA Extraction and Quantitative Reverse Transcription-Polymerase Chain Reaction (qRT-PCR) Analysis

Cellular RNA was isolated using the Total RNA Extraction kit (Solarbio Life Science, Beijing, China). The cDNA was synthesized from 1 μg of RNA using the High Capacity cDNA Reverse Transcription Kit (Applied Biosystems, Foster City, CA, USA) according to the instructions provided by the manufacturer. Real-time qRT-PCR was performed using POWER SYBR Green Master Mix (Applied Biosystems). The following primers were obtained from GENEWIZ (Suzhou, China): Human p53, forward 5'CCCAGGTCCAGATGAAG3' and reverse 5'CAGACGGAAACCGTAGC3'; Human p21, forward 5'AAGACCATGTGGACCTGT3' and reverse 5'GGTAGAAATCTGTCATGCTG 3'; and GAPDH, forward 5'ATCACCATCTTCCAGGAGCGA3' and reverse 5'GCCAGTGAGCTTCCCGTTCA3'. Real-time PCR analyses were performed in the CFX Connect^TM^ Real-Time System (Bio-Rad, Hercules, CA, USA). The housekeeping gene GAPDH was used for normalization. The expression of individual genes was calculated according to the 2^(-ΔΔCt)^ methods.

### Methylation-specific PCR

The cells were treated with 5-Aza at 2 µM for 96 h. The EZ DNA Methylation-Direct^TM^ kit (ZymoResearch, Irvine, CA, USA) was used for isulfate conversion and extraction of genomic DNA according to the instructions provided by the manufacturer. Briefly, 1 × 10^5^ cells were harvested and treated with proteinase K for digestion. Next, DNA conversion was conducted by treatment with isulfate, followed by DNA extraction. Finally, PCR was performed in 25 μl reaction volume using 200 ng of modified DNA. The MethPrimer design tool (http://www.urogene.org/methprimer2) was used to design specific primers to determine the methylated and unmethylated status of the promoter fragments. Sequences around the transcription start site (TSS) were preferentially amplified. The following primers were used to amplify: methylated p53 (sense 5'GTAGGTAGAAGATTTTCGGGA3', antisense 5' GCGAAATCTAATCCGAAATACG3', PCR product was 158 bp), unmethylated p53 (sense 5'GAGTAGGTAGAAGATTTTTGGGA3', antisense 5'CCACAAAATCTAATCCAAAATACAAC3', PCR product was 162 bp), methylated p21 (sense 5'TACGCGAGGTTTCGGGATC3', antisense 5'CCCTAATATACAACCGCCCCG3', PCR product was 174 bp), and unmethylated p21 (sense 5'GGATTGGTTGGTTTGTTGGAATTT3', antisense 5'ACAACCCTAATATACAACCACCCCA3', PCR product was 164 bp)^29^. The PCR was conducted at 98°C for 5 min, followed by 40 cycles at 98°C for 10 s, 60°C for 30 s, and 72°C for 30 s with a final extension step at 72°C for 5 min. The PCR program was performed in gradient PCR systems (Eppendorf, Hamburg, Germany). Non-methylated and methylated human DKO DNA (Zymo Research) were also treated with isulfate and used as negative and positive controls, respectively. In addition, double-distilled water was used as blank control in the PCR assay. The PCR products were analyzed by running 2% agarose gels, stained with ethidium bromide, and visualized with Tanon 2500 (Tanon, Shanghai, China).

### Bioinformatics and Statistical Analysis

Clinical data of p53 and p21 expression in CaP tissues were extracted from NCBI GEO profiles datasets and were analyzed as described previously 30. All the experiments were repeated in triplicate. Data were presented as the mean ± standard deviation. The levels of significance for comparisons between samples were determined using the Student's t-test. P-values<0.05 denoted statistically significant differences.

## Results

### 5-Aza Inhibited CaP Cell Proliferation

**Figure 1:**
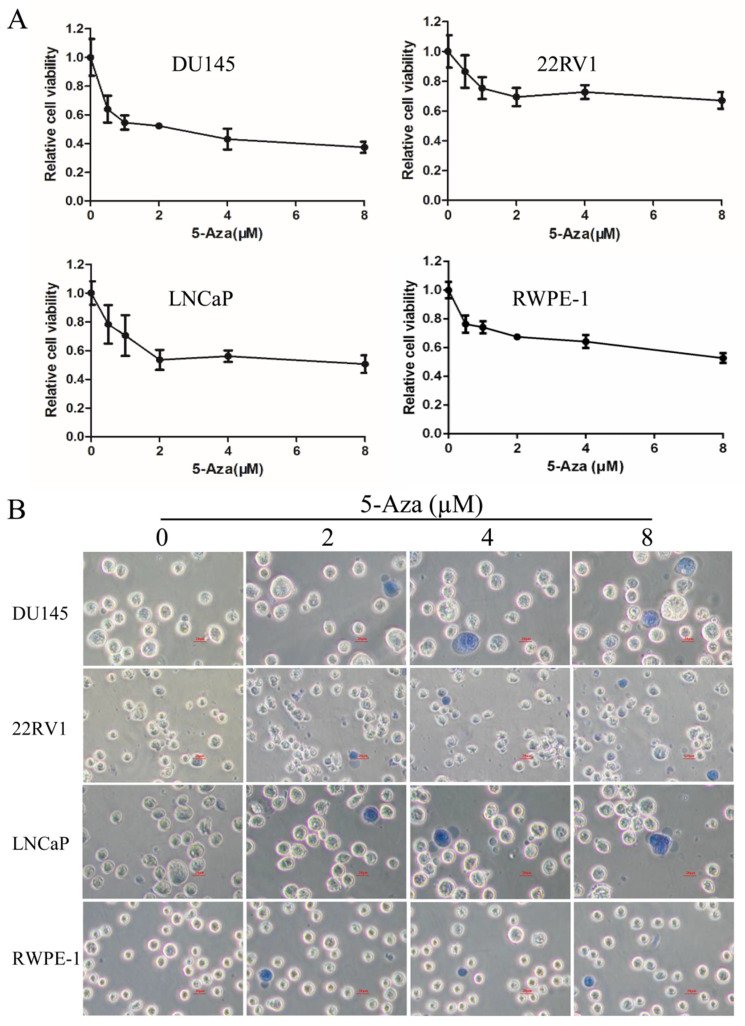
** 5-Aza inhibited the proliferation of prostate cancer cells. A)** DU145, 22RV1, LNCaP and RWPE-1 cells were treated with 5-Aza at 0.5-8 μM for 96 h. The cell viability was detected using a WST-1 assay. The data from each processing point represent the means ± SD of triplicate wells. Abbreviations: 5-Aza, 5-Aza-2′-deoxycytidine; SD, standard deviation. **B**) DU145, 22RV1, LNCaP and RWPE-1 cells were treated with 5-Aza at 2 µM, 4 µM and 8 µM for 96 h. The cell viability was detected using a trypan blue staining assay. The light microscopy was used to investigate the cell phenotype. Scal bar: 20µm. Abbreviations: 5-Aza, 5-Aza-2′-deoxycytidine.

In this study, RWPE-1 cell and human CaP cell lines DU145, 22RV1, and LNCaP were treated with 5-Aza at various concentrations for 96 h. Cell viability was detected by the WST-1 assay. The results showed that doses less than 2 µM of 5-Aza rapidly reduced the viability in all three types of CaP cells and RWPE-1 cell. However, this inhibitory effect of 5-Aza was gradually weakened or reached its plateau while using doses higher than 2 µM of 5-Aza (Figure 1A). When DU145 cells were treated with 5-Aza, the cell viability was rapidly decreased to 54.6% (1 μM) and subsequently slowly reduced to 37.3% (8 μM). A similar inhibitory pattern was also observed in LNCaP and 22RV1 cells. However, the inhibitory effect of 5-Aza on the proliferation of 22RV1 cell and RWPE-1 cell were significantly marginal (< 50%). Thus, treatment with 5-Aza impacted the cell proliferation differentially. Meanwhile, investigating the cell phenotype using light microscopy, we found a normal cell shape with refraction of light. There was no significant cell death with cell membrane damage (e.g., necrosis) found after performing the Trypan blue staining test (Figure 1B).

### 5-Aza Impacted the Cell Cycle Process in CaP Cells

**Figure 2:**
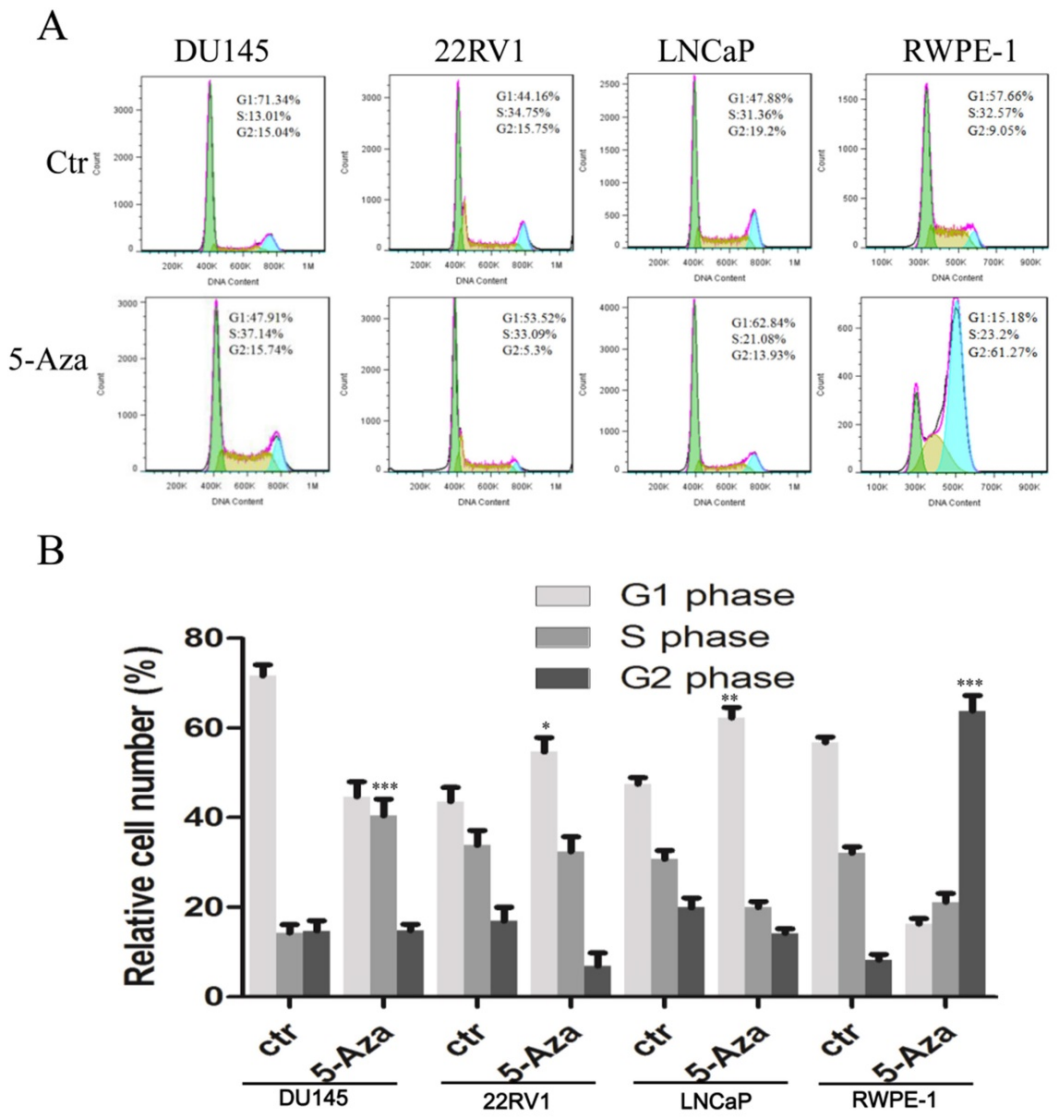
** 5-Aza impacted the cell cycle processin prostate cancer cells.** DU145, 22RV1, LNCaP and RWPE-1 cells were treated with 5-Aza at 2 μM for 96 h. The cells were harvested and the cell cycle status was analyzed by flow cytometry assay, as described in the Materials and Methods section. Data from one representative study are presented in (**A**), and data from three independent experiments are presented in (**B**). Note: compared with control group, * P<0.05, ** P<0.01, *** P<0.001. Abbreviations: 5-Aza, 5-Aza-2′-deoxycytidine; ctr, control.

To examine the effect of 5-Aza on the cell cycle process, CaP cells and normal RWPE-1 cell were treated with 2 μM of 5-Aza for 96 h, followed by staining with PI. Then, DNA contents in different cell cycle statuses were analyzed using flow cytometry assay. The results showed that, in a representative experiment, treatment with 5-Aza increased the accumulation of cells at the G1 phase from 44.16% to 53.52% in 22RV1 cell and from 47.88% to 62.84% in LNCaP cell. Interestingly, treatment with 5-Aza markedly increased the accumulation of DU145 cell at the S phase from 13.01% to 37.14%, but concurrently reduced the number of those at the G1 phase from 71.34% to 47.91%. Meanwhile, treatment with 5-Aza markedly increased the accumulation of normal immortalized RWPE-1 epithelial cell at the G2 phase from 9.05% to 61.27%, but reduced the portion of G1 phase cells from 57.66% to 15.18% (Figure 2A). These changes were confirmed in three independent experiments (Figure 2B). Thus, treatment with 5-Aza altered the cell cycle process differentially and arrested the cells at a certain phase.

### 5-Aza Induced DNA Damage and Apoptotic Cell Death

**Figure 3:**
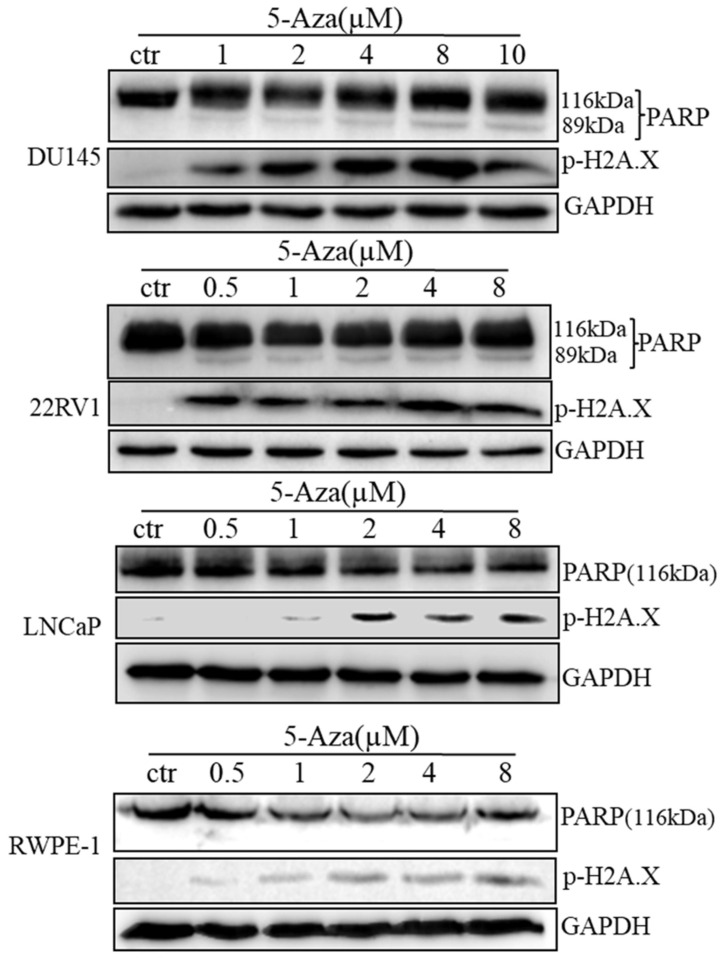
** 5-Aza induced phospho-histone H2A.X (Ser139) and PARP cleavage in prostate cancer cells.** Prostate cancer cells were treated with 5-Aza for 96 h at indicated concentrations ranging 1-10 μM in DU145, and 0.5-8 μM in 22RV1, LNCaP and RWPE-1 cells. The cells were harvested, followed by western blotting assay for the expression of apoptotic biomarker PARP cleavage and DNA damage biomarker p-H2A.X proteins. GAPDH was used as an internal loading control. Abbreviations: 5-Aza, 5-Aza-2′-deoxycytidine; ctr, control; GAPDH, glyceraldehyde-3-phosphate dehydrogenase; PARP, poly (ADP- ribose) polymerase.

**Figure 4:**
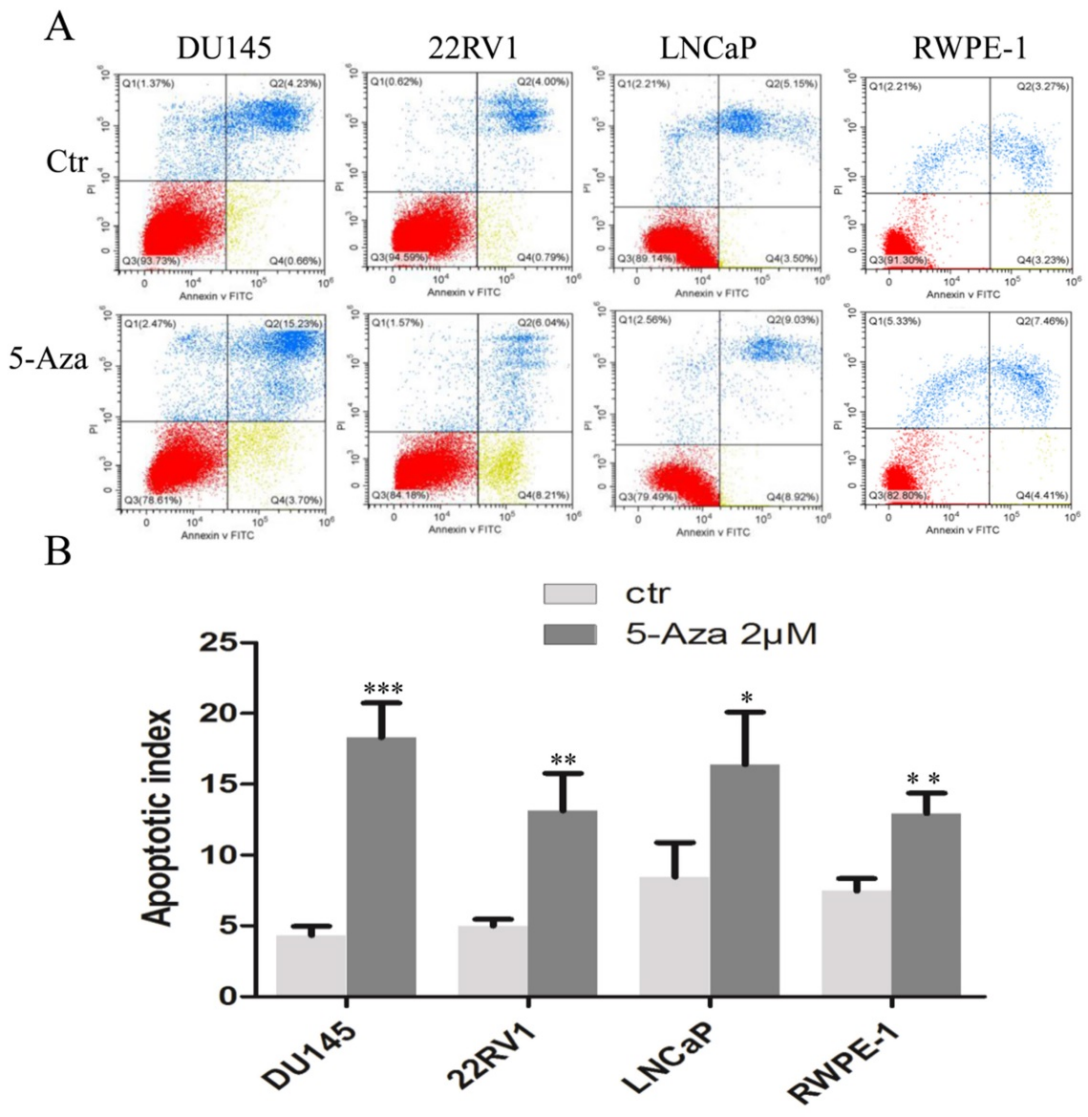
** 5-Aza induced apoptotic cell death in prostate cancer cells.** DU145, 22RV1, LNCaP and RWPE-1cells were treated with 5-Aza at 2 μM for 96 h. The cells were harvested and flow cytometry assay was used to analyze the apoptotic cell death, as described in the Materials and Methods section. One representative result is presented in (**A**). Data from three independent experiments are presented in (**B**). The bar denotes the mean ± SD. * P<0.05, ** P<0.01, *** *P*<0.001. Abbreviations: 5-Aza, 5-Aza-2′-deoxycytidine; ctr, control; FITC, fluorescein isothiocyanate; PI, propidium iodide; SD, standard deviation.

To clarify our previous finding of 5-Aza exerting differential cytotoxicity 30, the three CaP cell lines and RWPE-1 cells were treated with different concentrations of the agent for 96 h, followed by western blotting analysis for the expression of phospho-H2A.X (p-H2A.X) and PARP cleavage. The findings revealed that the levels of p-H2A.X protein were significantly and differentially increased after treatment of DU145, 22RV1, LNCaP cells and RWPE-1 cells with variant doses of 5-Aza (Figure 3), suggesting the presence of treatment-induced double strand DNA damage. Meanwhile, the apoptotic biomarker of PARP cleavage (89 kDa) was also observed and increased in DU145 cell, 22RV1 cell after treatment with 5-Aza, but it showed marginally in LNCaP cell and RWPE-1 cell. Interestingly, the levels of full-length PARP protein did not decrease in DU145 cell and 22RV1 cell, but it reduced in both LNCaP cell and RWPE-1 cell (Figure 3). Consistently, data from a representative study utilizing flow cytometry also showed that the percentage of apoptotic cells (Q2+Q4) with FITC Annexin V staining increased after treatment with 5-Aza from 4.89% to 18.93% in DU145 cells, from 4.79% to 14.25% in 22RV1 cells, from 8.65% to 17.95% in LNCaP cells, and from 6.50% to 11.87% in RWPE-1 cells (Figure 4A). These changes were confirmed and showed significance (for DU145, ***P<0.001. for 22RV1 and RWPE-1, **P<0.01. for LNCaP, *P<0.05) in three independent experiments (Figure 4B). Taken together, the results showed that 5-Aza induced more significantly apoptosis in aggressive androgen-independent CaP cells than in androgen-responsible LNCaP and normal prostatic epithelial RWPE-1 cell.

### 5-Aza Downregulated c-Myc Expression and Diminished Promoter Nuclear Acid Methylation of p53 and p21 Genes

**Figure 5:**
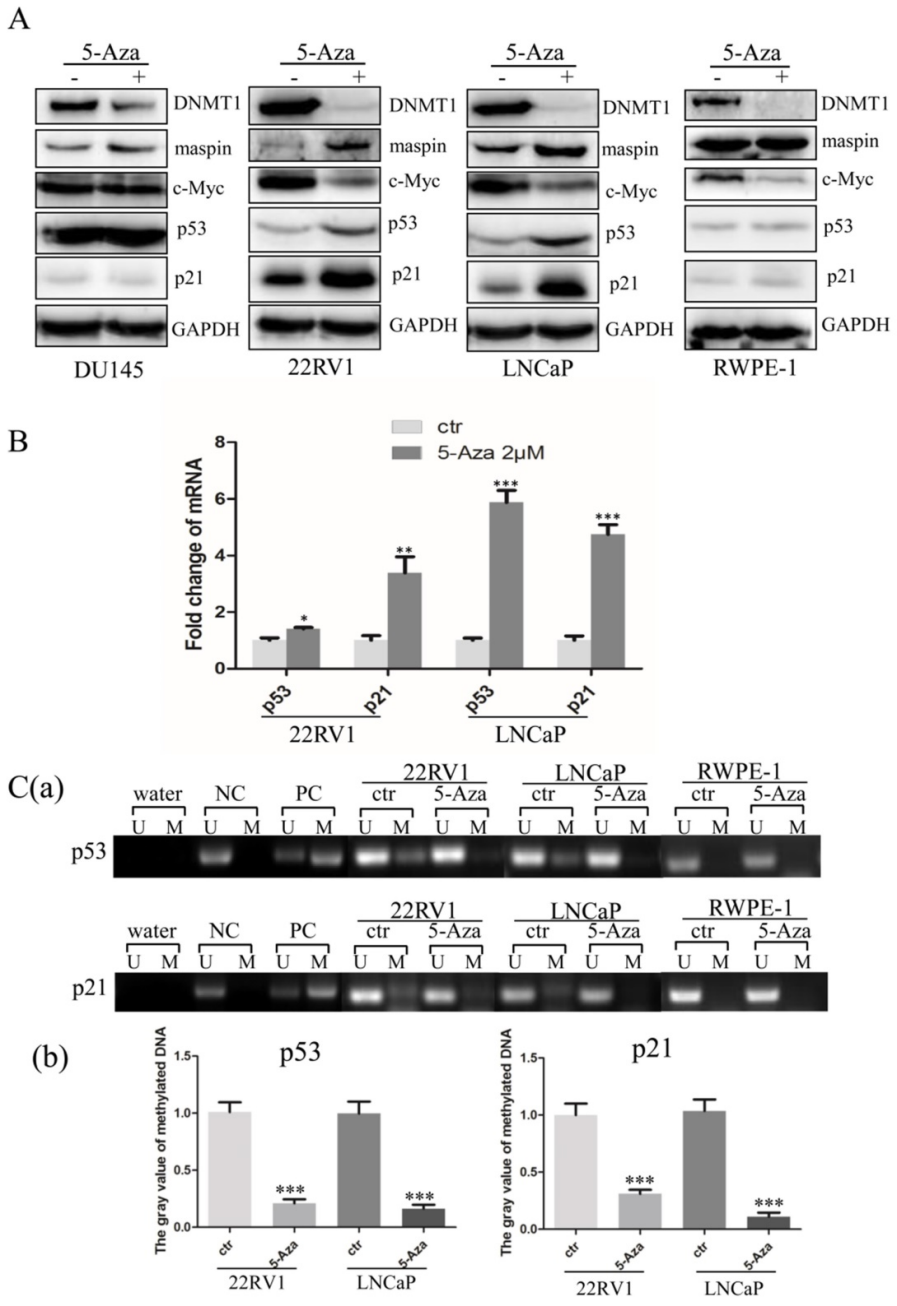
** 5-Aza activated p53 and p21 gene transcription and downregulated c-Myc expression in prostate cancer cells.** (**A**) DU145, 22RV1, LNCaP and RWPE-1cells were treated with 5-Aza at 2 μM for 96 h. The cells were harvested and the expression of DNMT1, maspin, c-Myc, p53 and p21 proteins was determined by western blotting. GAPDH was used as an internal loading control. (**B**) The 22RV1 and LNCaP were treated 5-Aza, as described above. The cells were harvested, the total cellular RNA was extracted, and qRT-PCR assay was performed to determine the mRNA levels of p53 and p21, as described in the Materials and Methods section. The bars denote the mean ± SD, * P<0.05, ** P<0.01, *** P<0.001. (**C**) 22RV1, LNCaP and RWPE-1 cells were treated with 5-Aza at 2 μM for 96 h. Next, the cellular genomic DNA was treated with sodium bisulfite to convert unmethylated cytosines to uracil. Methylation-specific PCR analysis was performed and the PCR products were separated on non-denaturing polyacrylamide gels. The bands reflecting methylated and unmethylated DNA of p53 (a) and p21 (b) were visualized by staining with ethidium bromide. ImageJ software was used to analyze the gray value of methylated DNA of p53 (c) and p21 (d). The bars denote the mean ± SD, *** P<0.001.Abbreviations: 5-Aza, 5-Aza-2′-deoxycytidine; ctr, control; DNMT1, DNA methyltransferase 1; GAPDH, glyceraldehyde-3-phosphate dehydrogenase; M: methylated; maspin, mammary serine protease inhibitor; NC, unmethylated negative control DNA; PARP, poly (ADP-ribose) polymerase; PC, methylated positive control DNA; qRT-PCR, quantitative reverse transcription-polymerase chain reaction; SD, standard deviation; U: unmethylated; Water, blank control of ddH_2_O.

To explore the molecular mechanisms through which 5-Aza inhibited cell proliferation with induction of apoptotic cell death, three CaP cells and RWPE-1 cells were treated with 5-Aza at 2 μM for 96 h, followed by western blotting analysis. As expected, treatment with 5-Aza induced maspin expression, which was accompanied by down regulation of DNMT1 in all cell lines 31-33. And tumor suppressive maspin was increased in all CaP cell lines. Meanwhile, following treatment with 5-Aza, the levels of c-Myc were significantly decreased, accompanied by a synchronous increase of maspin, p53 and p21 in 22RV1 and LNCaP cells. It was also noticed that down regulation of c-Myc and increase of p21 were found in normal RWPE-1 cells (Figure 5A), which was consistent with others report 34, 35. However, the above effects on expression of c-Myc, p21 and p53 were not observed in DU145 cells after treatment with 5-Aza (Figure 5A). Of note, data from the qRT-PCR analysis showed increased transcription of both p53 and p21 genes in both CaP epithelial 22RV1 and LNCaP cells after treatment with 5-Aza (Figure 5B). As 5-Aza is a demethylation agent, methylation-specific PCR was conducted to examine the methylation status of the promoter regions of p53 and p21 genes. The results revealed that increased methylation of the p53 and p21 gene promoters were seen in both CaP cell lines of 22RV1 and LNCaP, but not in normal RWPE-1 cells; however, methylation was significantly reduced or even undetectable after treatment with 5-Aza (Figure 5C) in CaP cells. Collectively, the above data suggested that treatment with 5-Aza inhibited DNMT1 expression, and prevented the methylation of the promoter nuclear acid to increase the transcription and expression of p53 and p21 genes in 22RV1 and LNCaP cells.

## Discussion

5-Aza (an HMA) has been used as a standard of care in patients with higher-risk myelodysplastic syndromes, and in those with acute myeloid leukemia ineligible for intensive therapy 36. Meanwhile, abnormal hypermethylation has been found to impact the expression of individual genes, and was associated with the malignant progression of solid cancer 37-39. However, the use of HMAs in patients with cancer has never achieved a satisfactory and beneficial overall outcome, particularly with regard to the genetic and epigenetic complexity, as well as heterogeneity 40, 41.

The present report revealed that 5-Aza inhibited the proliferation of CaP cells at a lower dose (< 2 µM); however, these inhibitory effects were mild to moderate (Figure 1). Moreover, 5-Aza-induced inhibitory effects reached a plateau at a higher dose (8 µM) and did not show significant cytotoxicity-induced cell membrane damage. The viability inhibition rather than cytotoxicity after treatment with 5-Aza differentially appeared from a lower to a higher degree in 22RV1, LNCaP, DU145 cells and the normal human prostate epithelial RWPE-1 (Figure 1). These 5-Aza-mediated antiproliferative effects also confirmed that the cell cycle was arrested at the S phase in DU145 cells, and at the G1 phase in 22RV1 and LNCaP cells after treatment with 5-Aza (Figure 2). Indeed, it was reported that 5-Aza inhibited growth by inducing cell cycle arrest at the G1 phase in multiple myeloma and various forms of lymphoma 42. In addition, 5-Aza also arrested the cell cycle at the S phase in human esophageal squamous cell carcinoma cell line TE-1^43^ and in mouse brain HT22 cells 44. Thus, collectively, the data suggested that the 5-Aza-induced differential cell cycle process in malignant cells was closely associated with the genetic and epigenetic heterogeneity of cancer. Since 5-Aza treatment showed modest inhibitory activities on CaP cells with certain degree of side effects on normal RWPE-1 cells, a better and selective treatment effects of 5-Aza on CaP could be expected if it was implemented in combination with other agents 30, 32.

Furthermore, treatment with 5-Aza induced immediately PARP cleavage (89 kDa) and p-H2A.X and its' level was kept steady in 22RV1 cells. Both PARP cleavage (89 kDa) and p-H2A.X were gradually increased along with the dose increase of 5-Aza in DU145 cells (Figure 3). This suggested that DU145 cells are more sensitive to 5-Aza-induced apoptosis than 22RV1 cells, which was consistent with the viability test results (Figure 1). DU145 cells expressed mutant p53 rather than wild type p53, which was expressed in 22RV1 and LNCaP cells. Hence, it could be hypothesized that these discrepancies in 5-Aza-induced DNA damage in different cells were, at least in part, due to the loss of p53-mediated repairing in DU145 cells. Although PARP cleavage occurred only in 22RV1 and DU145 cells, there was no significant increase in the PARP cleavage fragment (89 kDa) observed in parallel with the increase in the dose of 5-Aza. Accordingly, the full-length PARP molecule was not significantly decreased (Figure 3). In fact, a significant increase in DNA damage marker p-H2A.X was documented in all three CaP cell lines. Furthermore, 5-Aza-induced apoptotic events were more significant in DU145 cells, 22RV1 cells than in LNCaP cells and RWPE-1 cells (Figure 4). PARP is an enzyme and required for damaged nuclear acid reparation and genomic integrity. Both reduction of full-length of PARP and/or formation of 89 kDa fragment by caspase-3 lead to the loss of its enzymatic activity, therefore results to more DNA damage and/or apoptotic DNA degradation. In addition, the reduction of full-length PARP with no significant increase of PARP cleavage were usually reported and also showed in our 5-Aza-treated LNCaP and RWPE-1 cells, which indicated the further degradation-mediated disappear of 89 kDa fragment 45-47. The above data suggest that, apart from apoptosis, there were other mechanisms (e.g. 5-Aza-induced DNA damage, ferroptosis, etc) involved in 5-Aza-induced cytotoxicity 7-9. In addition of 5-Aza-induced cell cycle arrest at the different stage in variant CaP cells (Figure 2), our data indicate that multiple mechanisms are involved in the antiproliferative and cytotoxic effects of 5-Aza in CaP, including the induction of cell cycle arrest, apoptosis, and DNA damage.

In general, HMAs activates the expression of tumor-suppressive genes through promoter DNA demethylation 31, 32, antiproliferative activity, cell cycle arrest, and apoptosis 40, 44. Typically, 5- Aza-induced cytotoxicity has been associated with increased expression of p53, tumor necrosis factor- alpha, and 2'-5'-oligoadenylate synthetase 1 (OAS1) 48-50. In this study, treatment with 5-Aza increased the expression of the classic tumor suppressive p53, maspin, and cell cycle regulatory p21, and synchronously decreased that of oncogene c-Myc in both 22RV1 and LNCaP cells (Figures 5A, B). It was reported that tumor suppressive p53 and maspin repressed c-Myc activity through both transcriptional and posttranscriptional mechanisms, ultimately inhibiting cell growth 14, 51, 52. Thus, it was reasonable to hypothesize that tumor suppressive maspin and the p53/p21 signaling pathway are involved in 5-Aza-induced antitumor activity 44, 50.

5-aza-CdR-induced p53-mediated p21^Waf1/Cip1^ expression was dependent on either DNA damage or DNA demethylation 26, 27. However, down regulation of both p53 and p21 were usually observed in malignant tissue, including prostate cancer tissue/cells (supplementary data Figure 1) 53. In this study, 5-Aza increased significantly the expression of p21 in both CaP 22RV1 and LNCaP cells with wild type p53 expression, but not in DU145 cell with mutant p53 expression (Figure 5A) 54. It was notable that there was no methylation of p21 promoter in RWPE-1 cell (Figure 5C), which indicated that the increase of p21 expression was most likely due to 5-Aza-induced DNA damage followed by activating p53/p21-mediated repairing. This data was consistent with the Doig's report and suggested that the regulation equilibrium of p21 expression was required for the prostatic epithelial homeostatic function 55. Similarly, it was reported that treatment with 5-Aza induced p21 protein in a concentration-dependent manner in wild type p53-expressing HCT116 and RKO cells, but not in SW480 and HT29 cells with mutant p53 expression 56.

It was well documented that 5-Aza incorporated into both DNA and RNA during the nuclear acid synthesis process (S phase), which abrogated nuclear acid synthesis and arrested cell cycle in S phase 57. This phenomena also be seen in our data of 5-Aza-treated DU145 cell and in others reports 58, 59. However, it was also reported that both p53 and p21 were checkpoint molecules of G1 phase of cell cycle. In 22RV1 and LNCaP cells, treatment with 5-Aza increased both p53 and p21 companied with decrease of c-Myc, which could be proposed to inhibit CDK2 /cycline E activity and prevent the cell cycle process from G1 to S phase, resulted in cell cycle arrest in G1 phase 15. Meanwhile, it was reported that the cooperation of increase of p21 with decrease of c-Myc also inhibited CDK1/cyclin B activity and prevented the cell cycle process from G2 to M phase, arrested in G2 phase, which could be responsible for our 5-Aza-induced RWPE-1 cell cycle arrest in G2 phase (Figure 2). Taken together, our data demonstrated that treatment with 5-Aza impacted the cell cycle process differentially in terms of genetic variant of CaP tissue/cells.

It is thought that malignant tumors have genetic and epigenetic roots, and disruption of the equilibrium between oncogenes and tumor suppressor genes by epigenetic changes has been associated with the initiation and progression of different types of cancers 3. Although epi-drugs (e.g., HAMs) showed antiproliferative activity with mild toxicity, their application could impact chromatin conformation and alter global gene expression. Thus, based on other reports and our previous evidence 60, we believe that the identification of an appropriate biomarker (e.g. maspin, p53, p21, c-Myc) is desirable for the indication of clinical application of certain HMAs. This biomarker could predict the therapeutic benefit in the management of patients with CaP. In this study, increase of tumor suppressive maspin, upregulation of wild type p53-mediated p21 expression and decrease of oncogene c-Myc level could serve as the series of biomarker for 5-Aza-induced tumor cell apoptosis and DNA damagein androgen responsible 22RV1 and LNCaP cells. However, increasing maspin expression could be an only biomarker for evaluating 5-Aza treatment efficiency in androgen-independent DU145 cells. Actually, such efforts of identification of cancer biomarker for diagnostic purpose or for evaluation of therapeutic efficacy have taken place with continuous progress 48, 61.

## Supplementary Material

Supplementary figure.
