# Supplementary material for: The Differential Antitumor Activity of 5-Aza-2'-deoxycytidine in Prostate Cancer DU145, 22RV1, and LNCaP Cells
Source: J Cancer. 2021 Jul 25;12(18):5593–604. doi: 10.7150/jca.56709 (PMC8364635; doi:10.7150/jca.56709)
Supplement: Supplementary file 1 — Supplementary figure. [file jcav12p5593s1.pdf]

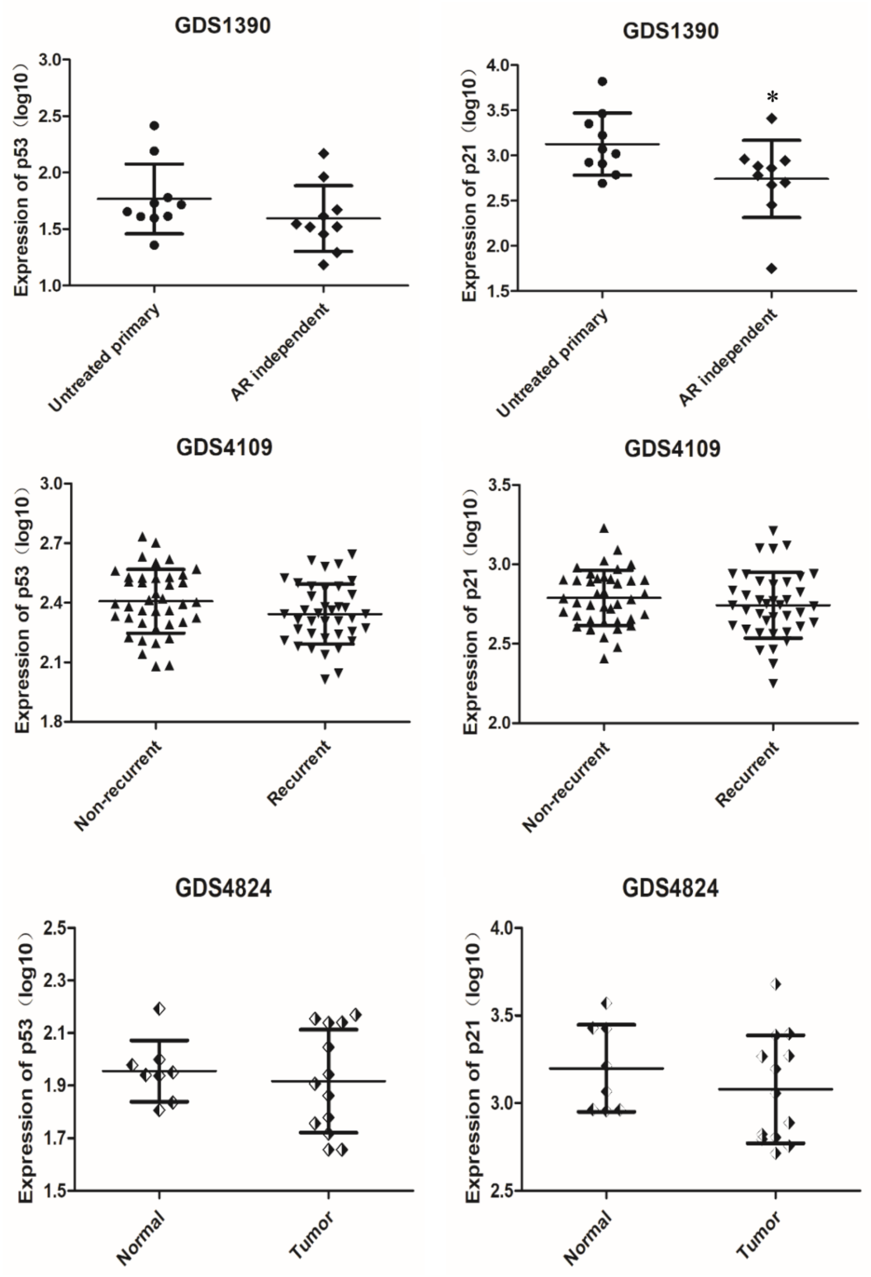

Supplementary Figure 1. Bioinformatics analysis of p53 and p21 expression in human prostate cancer tissues. Data were extracted from NCBI GEO profiles dataset. The bars denote the mean ±SD, * $P<0.05$.
